# Supplementary material for: Medical Interventions for Patellofemoral Pain and Patellofemoral Osteoarthritis: A Systematic Review
Source: J Clin Med. 2020 Oct 23;9(11):3397. doi: 10.3390/jcm9113397 (PMC7690719; doi:10.3390/jcm9113397)
Supplement: Supplementary file 1 [file jcm-09-03397-s001.pdf]

Supplementary Table 1. Individual study population and quality assessment of eligibility criteria (1 indicates quality indicator present, 0 not present).

First Author Year	Population	Inclusion items			Exclusion items				
**Pharmaceutical & Nutraceutical**		**Clear definition: symptom location**	**Clear definition: structural diagnosis**	**Clear definition: clinical diagnosis**	**History of trauma**	**History of dislocation**	**Evidence of subluxation or malalignment**	**History of surgery**	**Other reason for knee symptoms**
Hart 2019 [44]	PFP/CP (USA)	0	0	1	0	1	1	1	1
Nakhostin-Roohi 2016 [60]	women athletes with AKP (Iran)	0	0	0	0	0	0	0	1
Örsçelik 2015 [47]	Military with unilateral PFP (Turkey)	1	0	0	0	1	1	0	1
Singer 2011 [57]	AKP (Australia)	1	0	1	0	1	0	1	1
Marti-Bonmati 2009 [56]	degenerative patellar conditions (chondromalacia or OA) not requiring surgery (Spain)	0	1	0	1	1	0	0	1
Kannus 1992 & 1999 [58,59]	patients with PFP (Finland)	1 As above	0	1	0	0	0	0	1
Suter 1998 [49]	AKP, Sports Medicine clinic database (Canada)	1	0	0	1	1	0	1	1
Raatikainen 1990 [48]	CP patients who attended a Sports Medicine Clinic (Finland)	1	1	1	0	0	0	0	1
Fulkerson 1986 [55]	AKP (USA)	1	0	0	0	0	0	0	0
Antich 1986 [45]	Physical therapy patients with knee	0	0	0	0	0	0	0	0

	extensor mechanism disorders (USA)								
Bentley 1981 [54]	CP patients (England)	1	1	1	0	0	1	0	0
Matoso 1980 [46]	CP patients in hospital rheumatology department who had opted for medication instead of surgery (Switzerland)	0	1	1	0	0	0	0	0
Darracott 1973 [53]	PFP in military members attending Army Medical Rehabilitation Unit (England)	1	0	1	0	0	0	0	0
**Surgical**									
Joseph 2020 [43]	isolated PFOA (UK)	0	0	1	0	0	0	1	1
Odgaard 2018 [40]	isolated PFOA (Denmark)	0	1	0	0	0	1	0	1
Pagenstert 2012 [50]	patients with lateral patellar hypercompression syndrome (Switzerland)	1	0	1	0	1	1	0	1
Kettunen 2007 & 2012 [41,42]	hospital or outpatient clinic, PFP (Finland)	1	0	1	1	1	0	1	1
Owens 2002 [51]	recreational women athletes undergoing arthroscopy for isolated patellar	0	1	0	1	1	1	0	1

	cartilage lesions (USA)								
O’Neill 1997 [61]	athletic patients with AKP and lateral patellar tilting (USA)	1	0	1	0	1	1 sub/0 mal	0	1
Hejgaard 1982 [52]	patients admitted to hospital surgical department for CP (Denmark)	1	1	1	0	0	1	0	1

AKP = anterior knee pain; PFP = patellofemoral pain; OA = osteoarthritis; CP = chondromalacia patella; PFOA = patellofemoral osteoarthritis; sub = subluxation; mal = malalignment.

Supplementary Table 2. GRADE assessment for pain outcomes

Intervention	Study design	Risk of bias	Inconsistency	Indirectness	Imprecision	Publication bias	GRADE certainty
**Pharmaceutical & Nutraceutical** *Oral*							
**NSAIDS**	4 RCTs	One moderate RoB [54]<br>Three high RoB [49,55,56]<br>-1	No<br>0	No<br>0	CI contains meaningful ES, n<400<br>-1	Not tested (n<10)<br>0	**Low**
**Chloroquine**	1 RCT	High RoB [46]<br>-1	One study<br>-1	Pain outcome unknown validity/reliability<br>-1	CI not reported, n<400<br>-1	Not tested (n<10)<br>0	**Very low**
**Glucosamine**	1 RCT	High RoB [56]<br>-1	One study<br>-1	No<br>0	CI exceeds meaningful ES, n<400<br>-1	Not tested (n<10)<br>0	**Very low**
**Pharmaceutical & Nutraceutical** *Injections*							
**Glycosaminoglycan Polysulphate**	2 RCTs	Two High RoB [48,58,59]<br>-1	Yes<br>-1	No<br>0	CI contains meaningful ES, n<400<br>-1	Not tested (n<10)	**Very low**
**Hyaluronic acid**	1 RCT	Low RoB [44]<br>0	One study<br>-1	No<br>0	CI contains meaningful ES; <400 participants<br>-1	Not tested (n<10)	**Low**
**Platelet-rich plasma**	1 RCT	High RoB [47]<br>-1	One study<br>-1	Military; Dose is comparator<br>-1	CI spans meaningful ES, n<400<br>-1	Not tested (n<10)<br>0	**Very low**
**Botox**	1 RCT	Moderate RoB [57]<br>0	One study<br>-1	No<br>0	CI contains meaningful ES, n<400<br>-1	Not tested (n<10)<br>0	**Low**
**Anabolic steroids**	1 RCT	High RoB [53]<br>-1	One study<br>-1	Military only, 93% men<br>-1	CI exceeds meaningful ES, n<400<br>-1	Not tested (n<10)<br>0	**Very low**

**Pharmaceutical & Nutraceutical**<br>*Transdermal*							
**NSAIDs (phonophoresis)**	1 RCT	Moderate RoB [60]<br>0	One study<br>-1	Women athletes only<br>-1	Values not reported, n<400<br>-1	Not tested (n<10)<br>0	**Very low**
**Corticosteroids (iontophoresis, phonophoresis)**	1 RCT	High RoB [45]<br>-1	One study<br>-1	Vague diagnosis, sex not reported<br>-1	CI spans meaningful ES, n<400<br>-1	Not tested (n<10)<br>0	**Very low**
**Surgical**<br>*Arthroscopic*							
**Standard arthroscopy**	1 RCT	High RoB [41,42]<br>-1	One study<br>-1	No<br>0	CI spans meaningful ES, n<400<br>-1	Not tested (n<10)<br>0	**Very low**
**Radiofrequency debridement**	1 RCT	High RoB [51]<br>-1	One study<br>-1	Women only, isolated PF cartilage lesions<br>-1	CI exceeds meaningful ES, n<400<br>-1	Not tested (n<10)<br>0	**Very low**
**Surgical**<br>*Open surgeries*							
**Lateral retinacular surgery**	2 RCT	Two high RoB [50,61]<br>-1	No<br>0	Diagnosis specific to lateral patellar alignment or hypercompression<br>-1	CI contains meaningful ES, n<400<br>-1	Not tested (n<10)<br>0	**Very low**
**Anterior tibial tuberosity displacement**	1 RCT	High RoB [52]<br>-1	One study<br>-1	CP surgical candidates<br>0	CI exceeds meaningful ES, n<400<br>-1	Not tested (n<10)<br>0	**Very low**
**Patellofemoral arthroplasty**	2 RCT	One low RoB[40]<br>One moderate RoB[43]<br>0	Effects in different directions but not significant so results similar	Isolated PFOA<br>0	CI spans meaningful ES, n<400<br>-1	Not tested (n<10)<br>0	**Moderate**

0

RoB = risk of bias; RCT = randomized controlled trial; ES = effect size; PFOA = patellofemoral osteoarthritis; CI = confidence intervals

Decision guidelines for scoring: RoB: -1 for high RoB (low and moderate no reduction); Inconsistency: -1 if results differ among studies regarding significance or direction of effect; or if not enough evidence to assess (e.g. only 1 study); Indirectness: -1 if population characteristics limit generalizability; if comparator has unknown efficacy or if pain outcome is not a validated measure; Imprecision: -1 if sample size <400, or if confident intervals are wide and either contain clinically meaningful benefit (meaningful defined as SMD 0.5 or RR 25%), or span both clinically meaningful benefit and harm. Note if large ES (i.e. CI exceeds clinically meaningful difference), we only upgraded if study was also rated low RoB); Publication bias: only assess if at least 10 studies for every treatment comparison.
